# Comparing Proton Transfer Reaction (PTR) and Adduct Ionization Mechanism (AIM) for the Study of Volatile Organic Compounds

**DOI:** 10.3390/molecules31030402

**Published:** 2026-01-23

**Authors:** Sara Avesani, Bianca Bonato, Valentina Simonetti, Silvia Guerra, Laura Ravazzolo, Gabriela Gjinaj, Marco Dadda, Umberto Castiello

**Affiliations:** 1 Department of General Psychology, University of Padova, Via Venezia 8, 35131 Padova, PD, Italy; sara.avesani@unipd.it (S.A.); valentina.simonetti@unipd.it (V.S.); silvia.guerra@unipd.it (S.G.); gabriela.gjinaj@unipd.it (G.G.); marco.dadda@unipd.it (M.D.); umberto.castiello@unipd.it (U.C.); 2 Department of Agronomy, Food, Natural Resources, Animals and Environment (DAFNAE), University of Padova, Viale dell’Università, 16, 35020 Legnaro, PD, Italy; laura.ravazzolo@unipd.it

**Keywords:** proton transfer reactor, adduct ionization mechanism, ion-molecule reactor, volatile organic compounds, real-time detection, pea plant, *Pisum sativum*, legumes, pulse crop

## Abstract

Volatile organic compounds (VOCs) play a central role in plant communication and ecology, acting as a chemical language that mediates interactions with other organisms and responses to environmental stimuli. Analyzing changes in the plant volatilome enables the effective differentiation between biotic and abiotic stresses. Consequently, monitoring VOC emissions offers valuable insights into plant signaling pathways and health status. These insights position this approach as a promising strategy for improving crop protection. Direct infusion (DI) online analytical techniques, such as proton transfer reaction mass spectrometry (PTR-MS) and adduct ionization mechanism mass spectrometry (AIM-MS), have been developed to detect and characterize VOCs in real time. Here, we evaluated the suitability of PTR-MS and AIM-MS for monitoring VOC emissions in pea plants (*Pisum sativum* L.). Comparative analysis revealed that AIM-MS, a recently developed technology, detected a higher number of distinct signals than PTR-MS. Annotation of detected and significant AIM-MS signals indicated a predominance toward those that were putative lipids-derived and amino acids-derived, whereas PTR-MS signals were primarily associated with putative phenolic compounds. These findings suggest that the newly developed AIM reactor offers a broader detection range and may enhance our ability to monitor plant VOC emissions. Consequently, AIM-MS emerges as a promising tool for the real-time assessment of pea plant health and stress responses. Further efforts are needed to improve the portability of DI-MS techniques and to integrate them with GC-MS techniques. Overall, these efforts will allow this technology to be exploited for plant protection in compromised environments.

## 1. Introduction

Plants produce a huge amount of volatile organic compounds (VOCs), which are small molecules characterized by a low molecular weight and high vapor pressure that quickly evaporate in the atmosphere to reach their biological targets [1,2,3]. Due to their properties, VOCs are ideal signaling molecules for mediating both short- and long-distance interactions, and for playing essential ecological and biological roles [4,5]. They can be grouped into four chemical classes (i.e., terpenoids, phenylpropanoids and benzenoids, fatty acid derivatives, and amino acid derivatives), and may be either constitutive (i.e., continuously emitted) or induced (i.e., elicited by stresses or during specific developmental stages) [6]. The amount of constitutive and induced VOCs is influenced by biotic and abiotic factors that modulate plant emission dynamics [5]. VOCs produced by emitter plants are decrypted by receiver plants, functioning as a chemical communication for the activation of resistance mechanisms [6,7,8,9,10]. It follows that deciphering and continuously monitoring how plants perceive their neighbors’ messages could provide rapid information on plant health status [7,11,12,13,14,15,16]. This suggests the necessity to investigate VOCs with a key role in stress response, and which of them are reliable biomarkers, i.e., measurable indicators of a plant’s cellular response to external stimuli [17,18]. Continuous field monitoring of identified VOC biomarkers could serve as early warning signals to detect stresses before severe damage occurs [12,17,18,19]. For diagnostic applications, it was observed that the emission rate and type of VOCs by diseased plants are quite different from healthy controls [20,21,22,23]. While the correlation of disease status to a specific VOC is relatively weak, growing evidence has shown that a panel of VOCs with a particular composition could be used as effective diagnostic biomarkers [23]. The collective analysis of plant VOC emission profiles generates a set of multidimensional data, the so-called molecular fingerprint, for effective differentiation of stresses [23,24]. The intention of fingerprinting is not to identify each observed metabolite, but to compare patterns of volatiles that change in response to disease, toxin exposure, environmental or genetic alterations [18]. Although the comprehension of plant communication provides a promising avenue for real-time health status monitoring, the understanding of volatile emissions and perception needs to be consolidated [12].

The analytical approach for VOC evaluation is indicated with the term of volatilomics, a field of metabolomics targeted to the detection, characterization, and quantification of all volatile metabolites (volatilome) in a biological system [25]. Available technologies for the study of the plant volatilome involve mass spectrometry platforms that can be divided into two main groups, direct and indirect methods [18,19]. The indirect methods couple mass spectrometry (MS) with separation techniques, such as gas chromatography (GC) [18]. These approaches are extremely powerful in terms of detection, quantification, and identification of a wide range of VOCs [18], and are the most prevalent techniques employed for studying plant volatiles [8,26,27]. However, GC-MS is an offline method and it cannot detect plant VOCs in real time (i.e., online), given that requires a rather long analysis time for the chromatographic separation [18]. These limitations have been addressed in recent years by the development of direct methods, i.e., direct infusion (or injection) mass spectrometry (DI-MS) techniques, which do not involve a separation step [15,19].

DI-MS online techniques, such as proton transfer reaction (PTR-MS) and adduct ionization mechanism (AIM-MS), are characterized by soft chemical ionization conditions which limit the fragmentation of chemical compounds. When these ionization approaches are coupled with high-resolution mass analyzers, they provide high sensitivity and mass resolution (necessary for trace analysis and determination of the exact mass of the ion). These techniques also allow for easy sampling, and the possibility to conduct the measurement over an extended period of time [15,19]. In particular, soft ionization methods ensure minimal compound variation during the analysis, allowing easier interpretation of the final result [28,29,30]. The PTR-MS analytical techniques are capable of quantifying VOCs in complex gas mixtures, detecting VOCs in traces (parts per trillion), and tracking concentrations that rapidly change over time [30]. In PTR-MS, the neutral VOCs are ionized through a proton transfer reaction, typically using hydronium ions (H_3_O^+^), in an ion source region of the reactor [31]. Ions produced in the ion source enter the PTR-MS drift tube, where the gaseous samples are also injected, and the proton transfer reaction occurs. At the end of the drift tube, a mass analyzer detects the ionic products [28]. Nowadays, PTR-MS instruments have been coupled with systems that permit the switching from H_3_O^+^ to another positive ionizing reagent, such as NO^+^, O_2_^+^, Kr^+^, and Xe^+^, allowing the detection of compounds such as CO, CO_2_, or ethylene and acetylene that are not detectable using H_3_O^+^ [29]. A recent and promising technology based on the adduct ionization mechanism (AIM) enables sampled VOCs to be ionized via chemical ionization at medium pressures, further reducing the risk of alteration or fragmentation of sampled compounds [32]. Moreover, the AIM-MS presents low detection limits of parts per quadrillion, and it has the capability of measuring a wide range of gaseous organic and inorganic species with time resolutions of up to 50 Hz [32]. The Vocus AIM reactor supports the use of many ionizing reagents of both positive (i.e., benzene cations [C_6_H_6_^+^], acetone dimer ([C_3_H_6_O]_2_H^+^), and ammonium [NH_4_^+^]) and negative (i.e., chloride [Cl^−^], bromide [Br^−^], iodide [I^−^], and nitrate [NO_3_^−^]) polarity, and is largely independent of changes in sample humidity [32]. Reagent gases and sample flow enter directly into the center of the conical reactor, and their collision allows the formation of product ions [32]. At the exit of the AIM reactor, product ions are guided by a radio frequency (RF) quadrupole ion guide that efficiently focuses the analyte ions into a narrow beam toward the detector [32]. In sum, while PTR-MS is a well-established technique for detecting VOCs with relatively high proton affinity, it is limited in its ability to ionize compounds with lower proton affinities or those that do not readily undergo proton transfer. AIM-MS can complement PTR-MS by ionizing additional VOC classes that may be poorly detected by PTR-MS. A wide spectrum of hydrocarbons, reactive nitrogen species, organic compounds with varying volatilities, and inorganic acids can be detected by AIM-MS, with sensitivities often exceeding ∼10 counts s^−1^ pptv^−1^ per (10^6^ reagent ions). Although the sensitivity of the Vocus AIM reactor does not necessarily surpass that of earlier low- and medium-pressure reactor designs [32], it remains within a comparable range and achieves extremely low limits of detection, typically between 0.1 and 5 ppt for most analytes. Comparing the two methods allows us to assess differences in chemical coverage, providing insight into the strengths and limitations of each approach for untargeted plant VOC profiling.

In this study, we compared two DI-MS techniques, such as the PTR-MS and the AIM-MS in positive mode (using benzene as the reagent) under their respective optimal conditions, to determine which reactor is more effective for the fingerprinting of the pea plants (*Pisum sativum* L.) volatilome. The pulse crop *Pisum sativum* L. is of great interest because it presents the highest-protein food value among pulses, according to the nutrient-rich food index [33,34]. It is a significant source not only of essential nutrients but also of starch and proteins in the human diet [35,36], and in industry applications [37,38]. Additionally, the pea ranks third among the most cultivated legumes in the world, following soybeans and beans, with primary production occurring in temperate regions (FAOSTAT, 2024; Smýkal et al. [39,40]). To date, there is limited knowledge regarding VOC emissions in pea plants [41]; therefore, an accurate identification of pea VOCs using novel, reliable, and sensitive analytical methods is a timely and important issue.

## 2. Results

**Figure 1:**
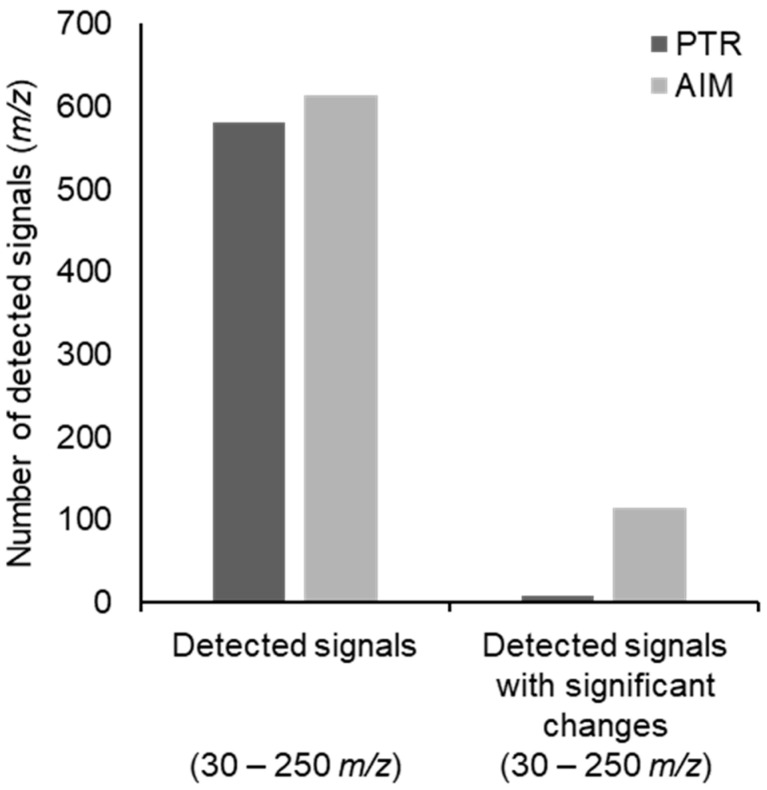
Graphical representation of the detected signals obtained with either PTR or AIM reactor. The absolute number and the number of signals with significant changes over time are reported.

A total of 581 and 614 *m*/*z* values were obtained with the PTR and AIM reactors, respectively, from the signals detected in the 30–250 *m*/*z* range (Figure 1, Supplemental Tables S1 and S2).

**Figure 2:**
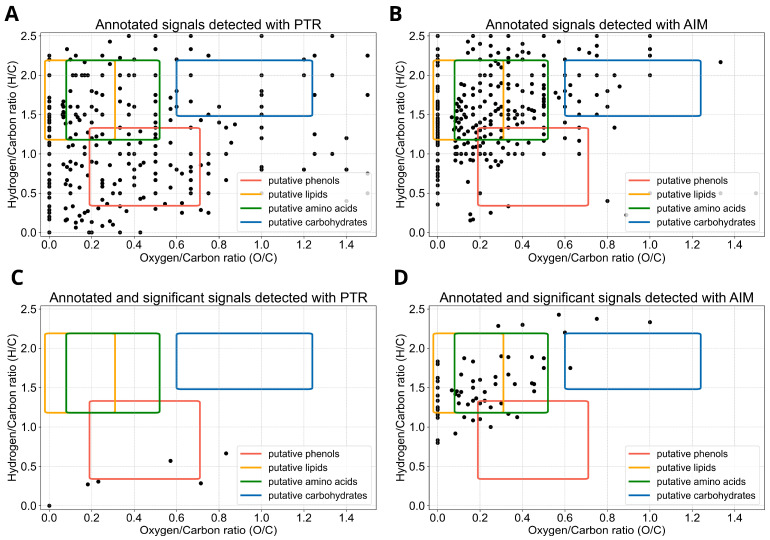
Van Krevelen diagram of annotated signals. The diagram is built according to hydrogen/carbon (H/C) and oxygen/carbon (O/C) ratios, and it indicates the putative chemical classes according to elemental formulas, such as putative carbohydrates (green), putative lipids (yellow), putative amino acids (blue), and putative phenols (red) for *m*/*z* value detected with PTR (**A**,**C**) or AIM (**B**,**D**) reactor.

**Figure 3:**
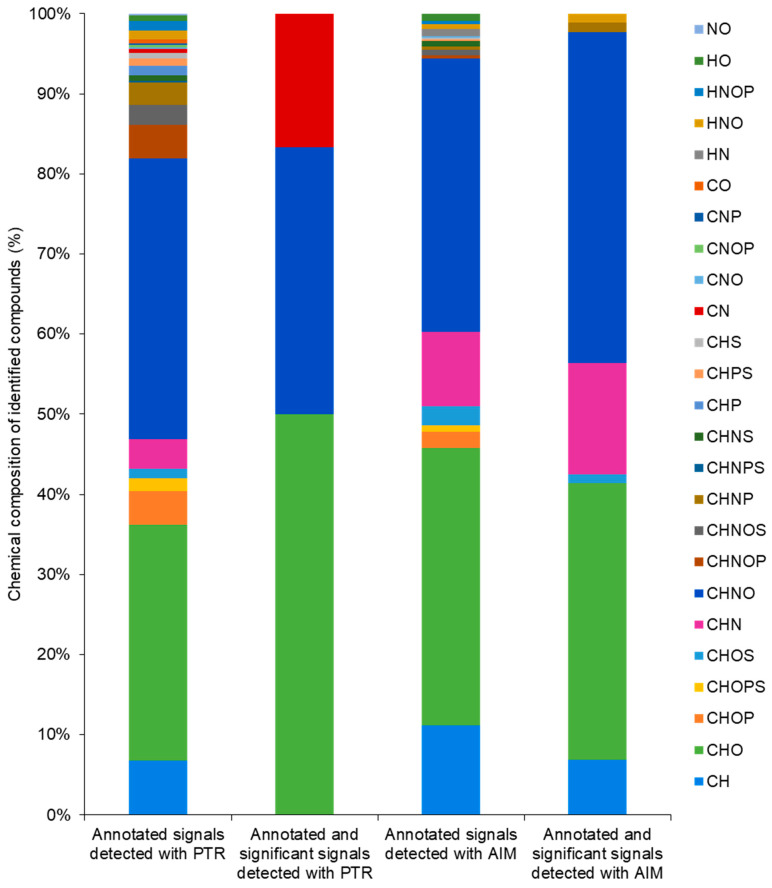
Graphical representation of the chemical composition of annotated signal. The frequency histogram representing the combinations of six chemical elements, carbon (C), hydrogen (H), oxygen (O), nitrogen (N), phosphorus (P), and sulfur (S), is built according to putative chemical formulas of annotated signal.

Detected signals with significant changes in time were obtained by applying the Mann–Whitney U test imposing an FDR-adjusted *p*-value lower than 0.05, and were 8 for the PTR and 114 for the AIM reactor (Figure 1; Supplemental Tables S3 and S4). Detected signals were annotated as previously indicated, and the assigned chemical formulas were used to plot H/C ratios against O/C ratios in a Van Krevelen diagram (Figure 2), and to build a frequency histogram of elemental compositions (Figure 3). The Van Krevelen diagram for the annotated signals detected with the PTR reactor exhibited a less dense distribution of signals for the putative lipids and amino acids area, but signals were more widespread throughout the entire diagram. In contrast, the Van Krevelen diagram for the annotated signals identified with the AIM reactor showed a greater concentration of signals in regions corresponding to potential lipids and amino acids (Figure 2A,B). Annotated and significant signals detected with the PTR reactor primarily belonged to the putative phenols chemical class, as they were mainly distributed in the region with H/C ratios lower than 1 (Figure 2C). In contrast, annotated signals detected with the AIM reactor primarily belonged to putative lipids-derived and amino acids-derived, as the highest density of chemical formulas showed H/C ratios ranging from 1 to 2, and O/C ratios ranging from 0 to 0.5 (Figure 2D). It is important to note that the Van Krevelen diagram was used to visualize the elemental composition space (H/C vs. O/C) of molecular formulae detected from our high-resolution MS data. These classifications (e.g., ‘putative lipids-derived’, ‘putative carbohydrate-derived’) refer to formula space only and do not imply detection of intact molecules. Gas-phase ions measured here may represent volatile fragments, derivatives, or thermal/ionization products; interpretations are therefore limited to trends in elemental composition rather than definitive structural identifications. Detailed identification of particular metabolites will be an extremely interesting subject for future studies.

It is important to note that the goal of this study was not to perform compound identification or quantification, but to compare the ability of the two technologies to explore the chemical space.

The reagent ion (derived from water and benzene) involved with the two reactors, the ionization reaction, and the reactor design, influence the selectivity of the two analytical techniques, leading to the detection of signals belonging to different chemical species (Figure 2).

The chemical composition of annotated signals showed the presence of ten major chemical species (i.e., CH, CHO, CHOP, CHOPS, CHOS, CHN, CHNO, CHNOP, CHNOS, CHNP) in both reactors, with comparable percentages exceeding 90%. Differences in detected and annotated species appeared in the others (Figure 3). Detected signals with significant changes showed a variation in chemical composition distribution. The PTR reactor allowed us to detect significant signals with a chemical composition of CN, CHO, CNHO and CH according to their elemental formulas (Figure 3 and Supplemental Table S3). Likewise, the AIM reactor enabled the detection of significant signals with a chemical composition of HNO, CHOS, CHO, CHNP, CHNO, CHN, and CH (Figure 3 and Supplemental Table S4). Annotated and significant signals mainly showed CHO and CHNO composition, with the percentage of 50.00 and 33.33 for PTR, and 34.48 and 41.38 for AIM, respectively (Figure 3).

## 3. Discussion

Plants emit VOCs in a constitutive and induced manner in response to biotic attacks or abiotic stresses, which makes the emissions (intensity and patterns) an attractive target for plant fingerprinting, as they can be used to monitor changes in the VOC profile. To date, no studies have been conducted to delineate pea fingerprinting. This work establishes the foundation for the fingerprinting of *Pisum sativum* plants through the application of DI-MS techniques. The aim is to compare two reactors, the more consolidated PTR with the recently developed AIM (using benzene as the reagent), and to determine which one is more effective in the fingerprinting of the pea volatilome. Differences in the amount of detected signals depending on the type of reactor used were evident. The AIM reactor exhibited a higher detection power, as suggested by a higher number of *m*/*z* values of detected signals and detected signals with significant changes. Likewise, annotated signals exhibited a higher density in the putative lipids-derived, amino acids-derived, and carbohydrate-derived regions of the Van Krevelen diagram, not confirmed for the putative phenol region, where an opposite trend appeared. Traditionally, PTR-MS has been employed to monitor VOCs belonging to the chemical class of lipids, including isoprene, monoterpenes, and green leaf volatiles [27,28,42]. In our PTR-TOF-MS data, signals attributed to putative lipids were largely less prevalent, which could reflect either the inherently low concentration of these compounds in our samples or limitations in the reactor’s sensitivity. Conversely, the detection of putative lipids in AIM-TOF-MS data suggests the higher sensitivity of the AIM reactor and its capability to detect molecules in an extremely low range of concentrations [32]. Also, the AIM reactor can detect monoterpene oxygenated radicals (i.e., chemical species with formula C_10_H_15_O_>3_ and C_10_H_17_O_>2;_ Riva et al. [32]), which are always located in the lipid region on the Van Krevelen diagram, but with higher values for the *x*-axis (Figure 2). Phenolic compounds constitute an important group of plant secondary metabolites that provide pea plants with special adaptive, defensive or survival strategies [43]. Phenols originate from the aromatic amino acid phenylalanine and are characterized by the presence in their structure of one or more benzene rings [2]. In pea plants, different organs accumulate a wide variety of phenolic compounds, including flavonoids, isoflavonoids, and phenolic acids, which can be converted into VOCs and released into the atmosphere, contributing to the plant’s aroma and its interactions with the environment [2,8,44]. Interestingly, we observed a higher amount of putative phenols in the PTR-TOF-MS data. In addition, using benzene as the reagent ion influenced the selectivity of our method: C_6_H_6_^+^ preferentially forms stable adducts with nonpolar or unsaturated VOCs via π–π or dispersion interactions, whereas polar oxygenated compounds may form less stable adducts. As a consequence, the observed chemical coverage under AIM-MS reflects reagent–ion-specific selectivity, and the broader compound diversity compared with PTR-MS should be interpreted in this context. Using alternative reagent ions could shift the detection bias toward different classes of VOCs.

Elemental composition analysis further clarifies the distinct detection strengths of each reactor. While frequency histograms showed similar signal detection capabilities overall, AIM uniquely identified more diverse elemental formulas, such as HNO, CH, CHOS, CHNP, and CHN, particularly for signals showing significant changes. PTR predominantly identified CHO formulas, while AIM identified more CHON formulas. These findings reinforce that while both systems are suitable for VOC fingerprinting, each exhibits specific strengths that align with different analytical objectives [23,28,29,30,32]. The choice of reactor, therefore, should be based on the type of investigation being conducted. In untargeted VOC analyses, a reactor that can detect a broad array of compounds is preferred [45]. The AIM reactor demonstrated this capability most effectively. However, combining both PTR and AIM systems could maximize VOC coverage and provide complementary chemical insights into plant emissions. For example, the *m*/*z* values of 124.0881 detected with AIM were putatively annotated as C_8_H_12_O and the *m*/*z* values of 171.1078 detected with PTR were putatively annotated as C_10_H_18_O_2_. These align with previous reports identifying C_8_H_12_O as the volatile aldehyde 3,5-octadien-2-one from pea cultivars Crécerelle and Firenza [46], and C_10_H_18_O_2_ as the ester trans-3-hexenyl butyrate from pea cultivar Aragorn [47], in non-stressed conditions. This concordance further underscores the expanded detection capabilities achieved by combining the AIM and PTR reactors. In particular, employing two complementary ionization systems enhances chemical coverage and improves the likelihood of detecting a broader spectrum of volatile compounds, thereby enabling a more comprehensive metabolomic assessment. However, while DI-MS provides valuable insight into real-time VOC emissions, it cannot alone confirm compound identities. In addition, the assignments are tentative and based on putative chemical classes inferred from fragmentation patterns or database matches, not on direct detection of the compounds. Therefore, complementary GC-MS analysis will be necessary to specify the sampled molecules, introducing analytical separation and validating results with authentic standards.

The application of online DI-MS techniques, particularly AIM-TOF-MS, offers powerful tools to advance our understanding of plant VOC emissions and their roles in communication and stress signaling [15,29,30,32]. While PTR-MS has an established track record, the AIM reactor represents a newer approach with promising capabilities. However, the literature on AIM-MS in plant science remains scarce, and further studies are needed to fully explore its potential. Given that current knowledge on pea plant emissions is rather limited but rapidly increasing [41], *Pisum sativum* L. presents a compelling model for further AIM reactor developments. The identification of VOCs and their mechanisms of action provides novel instruments for the development of pea protection strategies, useful for an agrifood system that is shifting toward more sustainable practices [48,49]. In this context, AIM-MS has the potential to function as an early-warning tool, capable of detecting the onset of pest or pathogen pressure, thereby aiding in the mitigation of crop loss.

Future applications should aim to integrate AIM-TOF-MS into precision agriculture frameworks. While current instruments require power and are relatively large, continued technological advances in miniaturization and system integration could enable near-field or in-field, real-time monitoring of plant health, allowing for immediate responses to VOC-based stress indicators. Further research is necessary to elucidate VOC roles in plant defense mechanisms, particularly for pulses like peas. Additionally, technical challenges such as system portability, data interpretation, and standardization must be addressed. Nonetheless, growing technical capabilities and mechanistic understanding are making comprehensive VOC monitoring increasingly feasible, paving the way for more resilient and responsive agricultural systems.

## 4. Materials and Methods

### 4.1. Biological Materials and Growth Conditions

**Figure 4:**
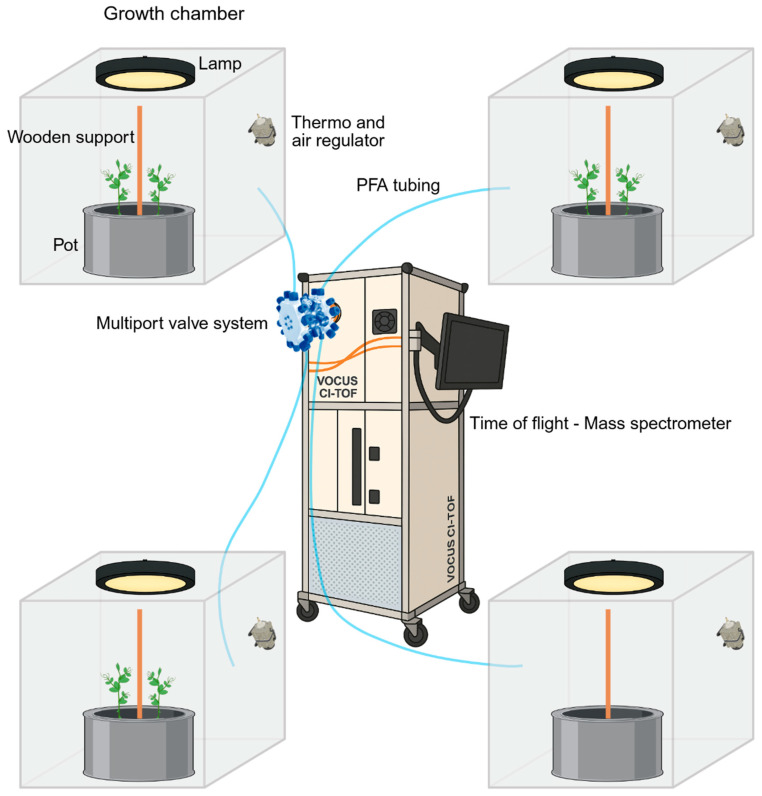
Graphical representation of the experimental set up representing the growth chambers and the TOF-MS, Vocus 2R.

Snow peas (*Pisum sativum* var. saccharatum cv Carouby de Maussane; Fratelli Ingegnoli spa, Milan, Italy) were rinsed for 1 h and a half in tap water. Rinsed seeds were germinated in darkness at 26 °C (FOC 200IL Connected Cooled Incubator, VELP^®^ SCIENTIFICA, Monza Brianza, Italy) in a filter paper strip (Whatman^®^ paper 3MM Chr sheets, Cytiva, Wilmington, NC, USA) soaked with demineralized water, 1.5 cm from each other and 0.5 cm from the top of the strip. Seed orientation showed the hilum and micropyle oriented downward. After four days of germination, pea seedlings were sorted, considering the most uniform for root and shoot length, and were planted in a custom-made phytotron. The phytotron (Esse Costruzioni srl, Frosinone, Italy) consists of a climatic room, in which temperature, humidity, fertilization, light, and air recirculation flow were set and controlled via a control panel. An air treatment unit consisting of air prefilter and filter, fan, and steam humidifier ensures the recirculation of purified air and the maintenance of set parameters. In the phytotron, growth chambers with a volume of 1 cubic meter are placed. Within each growth chamber, cylindrical pots (diameter 40 cm; height 20 cm) filled with silica sand (type 16SS, dimension 0.8–1.2 mm, weight 1.4) were placed (Figure 4). A slight overpressure (Δ 5 Pa from inside to outside) is ensured by a continuous flow of purified air.

Two seedlings were planted in each of the pots and were monitored for twelve days. To account for environmental differences across acquisitions, no seedlings were planted in one of the pots. The signal collected from the no-plants growth chamber was used as a baseline and subtracted from the signals obtained in the other chambers. All pots were watered and fertilized once every three days, using a half-strength solution culture (Murashige and Skoog Basal Salt Micronutrient Solution; 10×, liquid, plant cell culture tested; SIGMA Life Science, Milan, Italy) with microfiltered water. A support measuring 1.2 cm in diameter and 54 cm in height was placed at the center of each pot. Two seedlings were planted per pot, positioned 10 cm from the support and directly opposite each other (Figure 4). The seedlings were grown in controlled environmental conditions at a climatic room temperature of 24 °C ± 1.5 °C, 40 ± 10% relative humidity (RH), and with an 11.15 h photoperiod (5.45 am to 5 pm) under a cool white LED lamp (Q150W v2.0, PURE FACTORY, Granada, Spain) that was positioned 50 cm above each seedling. Photosynthetic Photon Flux Density of 200 μmolph/(m^2^s) was measured at 50 cm under the lamp (HD2302.0 photometer with PAR LP471PAR probe, Senseca, Padova, Italy) in correspondence with the seedling.

Six couples of plants were grown in total. For each MS modality, three biological replicates (couples of plants) were analyzed: three couples of plants were sampled for PTR-MS and three couples of plants for AIM-MS.

### 4.2. Volatile Organic Compounds Sampling and Analysis

VOCs were continuously sampled from the growth chambers using perfluoroalkoxy (PFA) Teflon tubing of 6 m length and 3/8 in. outer diameter (Figure 4). A manifold sampling system with a multiport valve (Tofwerk AG, Thun, Switzerland) ensured the connection of each tube emanating from the growth chambers to the time-of-flight mass spectrometer (TOF-MS, Vocus 2R; Tofwerk AG, Thun, Switzerland; Figure 4), which can hold two different reactors, a PTR, or an AIM ion–molecule reactor. The multiport valve system provided a valve switch every seven minutes, ensuring a minimum of one acquisition per hour from each growth chamber. At the exit of the Vocus reactor (either PTR or AIM), product ions are pumped into an RF-only quadrupole ion guide through a 1 mm orifice. The quadrupole ion guide focuses the analyte ions into a narrow beam, leading to the net removal of the neutral molecules by a vacuum pump (Ebara PDV 500, Tokyo, Japan). The quadrupole ion guide held at 10^−2^ mbar transfers energetically cooled ions into a lens stack held at 10^−5^ mbar before an orthogonal extraction in a TOF mass analyzer operated at <10^−6^ mbar. The instrument was configured to measure a mass-to-charge (*m*/*z*) ratio with a mass resolving power of 10,000–11,000 [32]. The acquired VOCs were analyzed in the range between 0 and 250 *m*/*z*. Data were recorded with a time resolution of 5 s and reported in Coordinated Universal Time (UTC). The PTR and AIM reactors were set to operate in their optimal working conditions; therefore, this study compares the two under their respective typical application settings rather than under strictly equivalent conditions.

#### 4.2.1. Volatile Organic Compounds Analysis with the PTR-TOF-MS

VOC analysis with the PTR-TOF-MS (Tofwerk AG, Thun, Switzerland) was carried out at a flow rate of 0.5 L/min (Defender 500 L, Mesa Labs, Munchen, Germany). Details for the Vocus PTR-TOF-MS are well described by Krechmer et al. [30]. Compared to the ionization in a conventional PTR-MS at 2.0–4.0 mbar, the Vocus ionization source is generally operated at low pressure [30]. Moreover, the Vocus chemical ionization source consists of a discharge reagent–ion source and focusing ion−molecule reactor (FIMR; Krechmer et al. [30]). The FIMR consists of a glass tube with a resistive coating, mounted inside an RF quadrupole. The axial electric field is used to enhance ion collision energies and limit cluster ion formation. The RF field focuses ions on the central axis of the reactor and improves the detection efficiency of product ions. Product ion signals are increased by a factor of 10 when the RF field is applied (5000−18,000 cps ppbv^−1^), improving measurement precision and detection limits while operating at very similar reaction conditions as traditional PTR instruments [30]. In the present study, H_3_O^+^ was chosen as the reagent ion at a flow of 0.02 L/min, and the following conditions were set: 100 °C Vocus PTR reactor temperature; 1.5 mbar Vocus PTR ionization source pressure; 350 and 400 V for axial and radial voltages Vocus PTR source voltage (Vocus reactor RF Amp in V). Sensitivity and mass calibration were carried out once a week using a VOC mixture of toluene, m-xylene, α-pinene, and 1,2,4-trimethylbenzene, at a concentration of 1 ppm and a flow of 0.005 L/min, diluted in ultra-high purity (UHP) N_2_.

#### 4.2.2. Volatile Organic Compounds Analysis with the AIM-TOF-MS

VOC analysis was carried out at a flow rate of 2 L/min (Defender 500 M, Mesa Labs) using the AIM-TOF-MS (Tofwerk AG, Thun, Switzerland), a new chemical reactor well described by Riva et al. [32]. The Vocus AIM reactor design includes improvements in sample and reagent ion introduction, a conductive polytetrafluoroethylene (cPTFE) Teflon^®^ conical reaction chamber to improve time response, and a simple yet efficient quadrupole-based, differentially pumped TOF interface [32]. In this work, the primary photoabsorbent benzene (C_6_H_6_^+^) at a flow rate of 0.25 L/min was chosen as the photoelectron source to generate the reagent ions, which lead to the formation of product ions colliding with sampled VOCs. Photoabsorbent benzene was selected as the reagent because of its high photoionization efficiency under the UV source used in this study and its good chemical stability over time. These properties ensure consistent ion generation and reproducible sensitivity, making benzene a reliable choice for AIM-MS applications targeting VOCs in plant emissions. In order to obtain photo-absorber benzene (C_6_H_6_^+^), gaseous benzene (C_6_H_6_, Sigma Aldrich, St. Louis, MO, USA, ≥99.9%) is maintained and delivered via a heated permeation tube, enters into a vacuum ultraviolet (VUV) lamp housing, and is photoionized, yielding C_6_H_6_^+^ and photoelectrons [50]. The instrumental conditions were set as follows: 80 °C permeation tubes; 50 °C Vocus AIM reactor temperature; and 50 mbar Vocus AIM ionization source pressure. Sensitivity and mass calibration were carried out once a week using a VOC mixture of benzene, toluene, m-xylene, α-pinene, 1,2,4-trimethylbenzene, at a concentration of 1 ppm and a flow of 0.01 L/min, diluted in UHP N_2_.

### 4.3. Data Processing, Statistical Analysis, and Annotation

**Figure 5:**
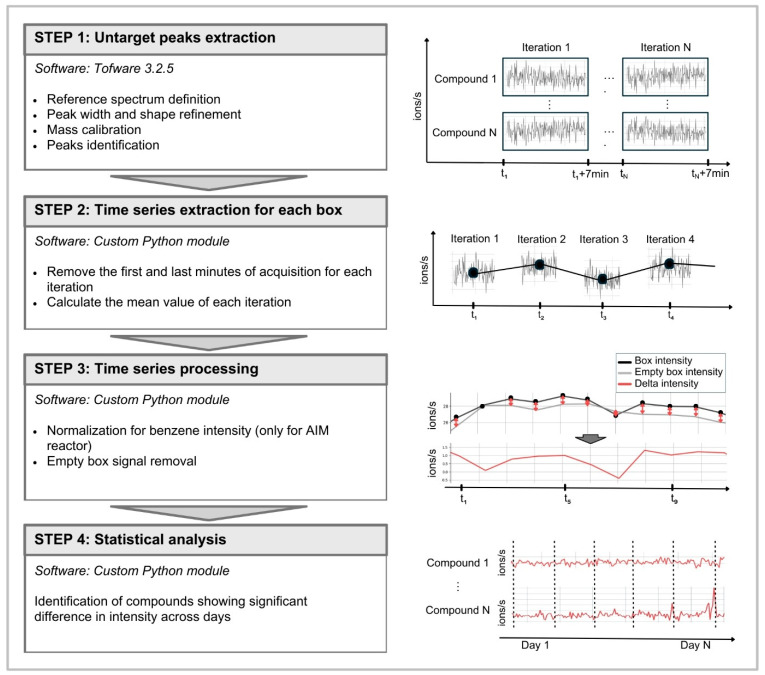
Data processing workflow applied to the collected data. The first step is the untargeted peaks extraction from raw data: it allows the time series extraction of each annotated signal in each processed file (different iterations corresponding to 7 min acquisition). The second step involves the time series extraction for each growth chamber: all the time series corresponding to different iterations are unified in a single time series where each time point corresponds to the average value for each iteration. The third step is the time series processing: all the time series obtained are prepared for the statistical analysis. Each signal intensity is normalized for the benzene intensity at each time point, and the corresponding intensity calculated inside the empty growth chamber is subtracted to account for possible environmental fluctuations. The last step is the statistical analysis: different days of acquisition are compared to identify compounds showing significant changes in their intensity. For this study, we compared day 8 to day 12 of acquisition.

Raw data were pre-processed and signals were extracted using an untargeted metabolomics workflow (twV3_2_5_wkf_Vocus_nonTargetedAnalysis_r4) in the Tofware software (Version 3.2.5; Tofwerk) based on the Igor software (Version 9.05 64-bit; WaveMetrics; Figure 5).

The pre-processing of raw .h5 files was carried out using “Campaign mode”, following the workflow steps: (i) reference spectrum definition, (ii) peak width and shape refinement, (iii) mass calibration, (iv) peaks identification in a range of *m*/*z* from 30 to 250, and (v) data export. The output of this processing step were .csv files containing data for each iteration (7 min acquisition with 0.5Hz sampling rate) in which each row corresponds to a time point and each column represents the intensity of the detected signal, i.e., *m*/*z* value. Files obtained from the peak extraction step were unified with a custom Python module (Python 3.12.3 version) to obtain a single time series for each detected signal for each growth chamber (Figure 5). For each iteration, we removed the first 2 min of acquisition to account for the transition between growth chambers, ensuring that the air from the previous iteration was not included in the analysis of the current iteration. We also removed the last 1 min of acquisition to account for possible effects of the multiport valve system switch. We then computed the average value of each iteration and appended them to create a complete time series for each annotated signal for each growth chamber. The obtained time series were saved as .csv files in which each row corresponds to a time point (one time point every 56 min) and each column represents the average *m*/*z* value of the detected signal for that iteration. This processing step was applied to correct for possible instrumental and environmental changes. For the AIM reactor, the obtained instrument signal is determined by the amount of the compound in the sample, but also by the reagent ion intensity. Considering this, we compensated for potential changes in the obtained instrument signal associated with changes in the reagent ion intensity due to the reagent supply consumption. This compensation was carried out with a second custom Python module (Python 3.12.3 version) by normalizing the intensity of each signal at a specific time point by the intensity of the benzene at the same time point. After the normalization for benzene concentration, we accounted for possible variations in unpredictable environmental conditions by subtracting from the detected signal intensity at each time point the corresponding intensity obtained in the empty growth chamber. Data handling and processing were performed using Python libraries pandas 2.2.2 and numpy 1.26.4.

The molecular formulas of detected signals were annotated using the Composition finder tool of the Tofware software (Version 3.2.5; Tofwerk) and the online tool ChemCalc (https://www.chemcalc.org/mf-finder; accessed on 20 January 2026). Different results were compared, and the most probable chemical formula was chosen with a ppm minor of 10. It will be necessary to determine the elemental formulas based on more precise mass measurements with lower error in future studies. Molecular formula annotations are tentative; they are software-generated assignments based exclusively on accurate mass. They should not be interpreted as definitive molecular identifications. Putative chemical classes of annotated signals were obtained with the van Krevelen diagram, according to the hydrogen/carbon (H/C) and oxygen/carbon (O/C) ratios of the elemental formula, such as putative carbohydrates-derived (O/C from 0.6 to 1.2 and H/C from 1.5 to 2.2), putative lipids-derived (O/C from 0 to 0.3 and H/C from 1.3 to 2.2), putative amino acids-derived (O/C from 0.1 to 0.5 and H/C from 1.3 to 2.2) and putative phenols (O/C from 0.2 to 0.7 and H/C from 0.4 to 1.4) according to Avesani et al. [51]. On the *x*-axis, the O/C ratio of the chemical formulas separates the compounds according to oxidation, whereas on the *y*-axis, the H/C ratio allows separation according to saturation [52,53]. A frequency histogram of possible combinations of six chemical elements, such as carbon (C), hydrogen (H), oxygen (O), nitrogen (N), phosphorus (P), and sulfur (S), was built according to the putative elemental composition of the elemental formula of annotated signals [51,54].

Statistical analysis was performed on data collected from day 8 to day 12 of acquisition according to the monitored plant growth. Starting from day 8, the plants exhibited a change in growth behavior, possibly associated with a change in VOC emission. Data for each day of acquisition and for each growth chamber were compared to identify which compound showed significant differences in its concentration across different days. Pairwise comparisons were performed using two-sided Mann–Whitney U tests (Python library scipy 1.16.1). Resulting *p*-values were adjusted for multiple testing using false discovery rate (FDR) (Python library statsmodels 0.14.1), with a significance threshold of FDR-adjusted *p*-value < 0.05. Only VOC features that remained statistically significant and reproducible across all biological replicates were retained.

## 5. Conclusions

This study demonstrated the capability of both PTR and AIM reactors to acquire and analyze signals emitted by pea plants during early growth stages under controlled, non-stressed conditions. Both systems enabled the detection of a diverse range of putative compounds, including lipids-derived, amino acids-derived, carbohydrates-derived, and phenols, though the specific signals and compound classes varied between the two techniques. The elemental composition of annotated signals confirmed the ability of both reactors to detect oxygenated and saturated molecules, highlighting their potential application in pea volatilome fingerprinting. It was observed that PTR is more suitable if the research objective focuses on monitoring phenolics. However, if the objective is wider in scope and includes amino acid derivatives, AIM may be advantageous. While further validation using complementary techniques, such as GC-MS, is necessary for definitive compound identification and deeper chemical characterization, the AIM reactor provided a broader spectrum of detectable signals compared to PTR. This suggests that AIM-TOF-MS holds promise as a tool for real-time, non-invasive monitoring of plant metabolic and health status. Continued development and application of this technology could significantly advance plant diagnostics and VOC fingerprinting, allowing for the resolution of important unanswered questions in plant chemical ecology, which, due to the lack of analytical time resolution, could not be answered in the past.

## Data Availability

The raw data supporting the conclusions of this article will be made available by the authors upon the acceptance of the article at the ZENODO repository.

## Supplementary Materials

The following supporting information can be downloaded at: https://www.mdpi.com/article/10.3390/molecules31030402/s1.
